# Sending Out Alarms: A Perspective on Intercellular Communications in Insect Antiviral Immune Response

**DOI:** 10.3389/fimmu.2021.613729

**Published:** 2021-02-23

**Authors:** Fei Wang

**Affiliations:** ^1^ State Key Laboratory of Silkworm Genome Biology, Southwest University, Chongqing, China; ^2^ Biological Science Research Center, Southwest University, Chongqing, China

**Keywords:** insect, antiviral immunity, cytokine, Dicer-2, intercellular transfer

## Abstract

Viral infection triggers insect immune response, including RNA interference, apoptosis and autophagy, and profoundly changes the gene expression profiles in infected cells. Although intracellular degradation is crucial for restricting viral infection, intercellular communication is required to mount a robust systemic immune response. This review focuses on recent advances in understanding the intercellular communications in insect antiviral immunity, including protein-based and virus-derived RNA based cell-cell communications, with emphasis on the signaling pathway that induces the production of the potential cytokines. The prospects and challenges of future work are also discussed.

## Introduction

Viral infection has posed a significant threat to human and animal health, agricultural production and environmental safety. The frequent outbreaks of pandemics caused by viral infection taught us bitter lessons that the long-standing battles between the hosts and viruses are much rougher than expected. As obligate intracellular pathogens, viruses heavily rely on the host cell machinery and resources to replicate and propagate. Accordingly, host cells develop multiple strategies, including intrinsic antiviral response that directly restricts viral replication and assembly, and induced antiviral response that potentiates the antiviral activity of viral-restricting factors or cells to suppress and eliminate the invading pathogens (1–4).

Insects are the most abundant and diverse group of animals in the world. Some of them are regarded as model organisms, disease vectors, agriculture and household pests or industrial animals. A lot of studies have been carried out to investigate molecules, pathways and mechanisms that are involved in the immune response of different insects upon viral challenges. Among them, a few attentions are given to how extracellular signaling networks cooperate with intracellular pathways to mount a robust systemic immune response. Pieces of evidence have proposed that intricate intercellular communications occur in response to viral infection in insects, and helped us better understand the insect antiviral immunity in a systematic way.

The best characterized antiviral immune response in insects is RNA interference (RNAi) (3, 5). Three RNAi pathways have been identified in insects, including the small interfering RNA (siRNA) pathway, the microRNA (miRNA) pathway and the (PIWI-interacting RNA) piRNA pathway. Among them, siRNA has been most intensively studied as a potent antiviral defense strategy. siRNA is initiated by recognition and cleavage of double-stranded RNA (dsRNA) produced either as viral replication intermediate or as base-pairing viral transcript by Dicer-2 in host cells. Dicer-2, an RNase III family endonuclease, processes dsRNA into 19-23-nucleotide (nt) long siRNA duplex, which is subsequently loaded onto Argonaute-2 (Ago-2) endonuclease and integrated into a multiple protein complex, RNA-induced silencing complex (RISC). siRNA duplex is then unwound to generate the guide strand, which targets viral mRNA or genomic RNA containing complementary sequence for degradation through the RNase activity of Ago-2, thereby restricting viral infection. miRNA pathway was previously charactered in post-transcriptional regulation of gene expression during development, in which a 22-nt duplex miRNA processed by RNase III enzyme Drosha and Dicer-1 sequentially forms miRNA programmed RNA induced silencing complex (miRISC) with Ago-1 protein. Recently, both virus derived miRNAs that regulate insect gene expression and insect-encoded miRNAs that target virus mRNA were reported, highlighting its role in host-virus interaction (6, 7). The antiviral role of piRNA which commonly involves in genomic control of transposable elements is controversial in *Drosophila* (8, 9), while in mosquito piRNAs that are derived from acquired viral cDNA with the characteristic size range of 24–30 nt and features of ping-pong amplification cycle were discovered to specifically inhibit viral replication (10, 11).

Besides RNAi, viral-induced apoptosis and autophagy also play important roles in restricting viral infection (12, 13). The expression level of several pro-apoptotic genes, such as *reaper*, *hid*, and *p53*, increased in response to virus-induced stress, while anti-apoptotic genes, such as *diap1* decreased, resulting in onset of apoptosis and subsequent phagocytosis of viral-infected cells by haemocytes (14–17). Interestingly, sometimes this antiviral apoptosis is suppressed by host protein, as evidence found in silkworm that peptidoglycan recognition protein (PGRP) 2-2, inhibited baculovirus-induced apoptosis *via* Akt activation, reflecting arms race between insect and virus (18, 19). Recently, a few studies found autophagy occurs after *Drosophila* infected with vesicular stomatitis virus (VSV), Rift Valley fever virus (RVFV) or Zika virus as evidenced by the elevation of lipidated Atg8 (Atg8-II) level and accumulation of Atg8 in autophagic punctae (20–24). Silencing core autophagy genes, such as *atg5* or *atg8*, led to significant increase of viral load. Plasma membrane receptor Toll-7 has also been demonstrated to activate autophagy upon sensing VSV glycoproteins or RVFV (24, 25), which is independent of transcription factor NF-κB, whereas eliminating Zika virus by autophagy in *Drosophila* appears to be NF-κB-dependent (23).

In addition, genome-wide RNAi screening and transcriptional profiling has revealed a plethora of genes involved in antiviral immune response. Some of them have broad antiviral activity. For instance, negative elongation factor (NELF) and positive elongation factor b (P-TEFb) collaboratively mediate transcriptional pausing to potentiate the rapid activation of some inducible genes and are required to restrict viral replication in adult flies and mosquito cells (26). Some have been reported to be involved in anti-microbial immunity with uncharacterized antiviral activity. For instance, two anti-microbial peptide (AMP) coding genes, *diptericinB* and *attacinC* were up-regulated in transgenic flies expressing a Sindbis virus (SINV) replicon (27). Knocking-down their expression led to a modest but significant increase in SINV load, confirming their antiviral functions. In mosquito cells, Dengue virus (DENV) infection up-regulated the expression of a cecropin-like peptide which does not only have anti-bacterial activity, but also have anti-DENV and anti-Chikungunya virus activity (28). The enhanced expression of *gloverin*, *lebocin*, *attacin* was also observed in silkworm larvae infected with *Bombyx mori* nucleopolyhedrovirus (BmNPV) (29). Based on these facts, the Toll and IMD pathways, which are the two canonical NF-κB pathways responsible for immune response against bacterial and fungi infection, are considered to be implicated in anti-viral immunity (30, 31). But most of viral-induced genes remain enigmas in terms of the molecular mechanism underling their antiviral activity. For instance, virus-induced RNA 1 (*vir-1*), a marker of the induction of anti-viral response, is mainly regulated by JAK/STAT pathway (32). Loss of function of JAK (named Hopscotch in *Drosophila*) caused decreased expression of *vir-1*, increased viral load and decreased survival after *Drosophila* C virus (DCV) infection. However, the molecular mechanism of antiviral activity of Vir-1 is unknown.

## Intercellular Communications

**Figure 1:**
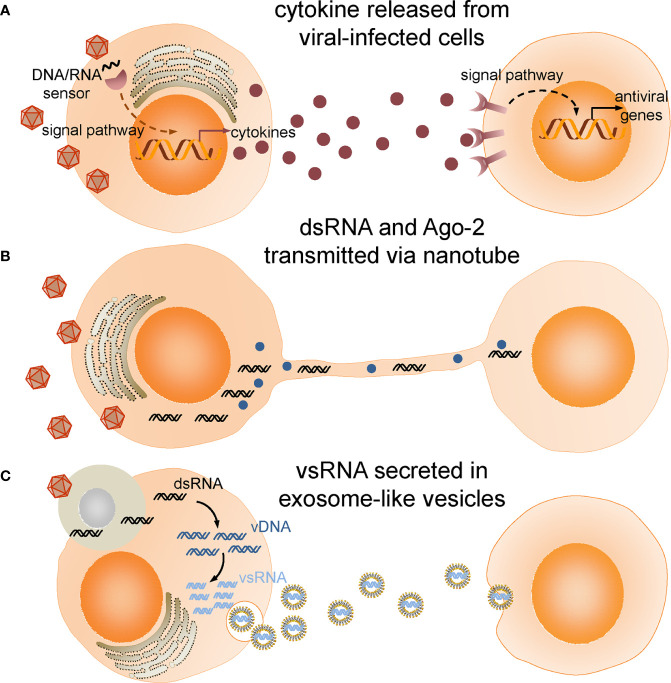
Intercellular communications in insect antiviral immune response. **(A)** Cytokines produced and released from viral infected cells bind to receptors and activate antiviral immune response in target cells. **(B)** Double-stranded RNA (dsRNA) and Ago-2 is transferred through tunneling nanotubes bridging infected cells and neighboring cells. **(C)** Viral-derived dsRNAs (vsRNAs) produced in viral-infected cells engulfed by haemocytes are reverse-transcribed into vDNAs by endogenous transposon reverse transcriptase. vDNAs then serve as template for transcription of secondary vsRNAs which are secreted in exosome-like vesicles and processed into siRNA by cells taking up these vesicles.

Although intracellular degradation is crucial to virus elimination, intercellular communication is believed to orchestrate and coordinate the cellular events. In the following, we will review the recent studies on extracellular signaling networks during antiviral immune response (**Figure 1**) and discuss the prospects and challenges of future work.

### Protein Based Intercellular Communication: Cytokines

As a comparison, the potent antiviral immune response in mammalian cells is largely dependent on a group of secretory protein collectively named cytokines, which are produced and secreted by viral-infected cells, and bind specific receptors on its own, neighboring or distant cells to initiate intracellular signaling mainly *via* JAK/STAT pathway (33, 34). Cytokines are divided into several subgroups, including interferon, chemokine, interleukin and tumor necrosis factor, among which interferon is particularly important for the immune response to virus. Cells activated by interferon synthesize various molecules that inhibit virus entry, replication and assembly, or produce inflammatory reactions to initiate apoptosis, autophagy and necrosis (35). Although comparative genomic analysis and evolutionary study revealed that insects do not possess the homologous molecules to vertebrate cytokines, the core components of JAK/STAT pathway including Hopscotch (JAK), STAT92E (STAT), negative regulators SOCS and PIAS have been identified in insects, and parallels between gain-of-function studies with mammalian homologs suggests the functional similarity of insect JAK/STAT pathway to vertebrates. Thanks to the genetic analysis of mutants defective in embryonic development, ligands and receptor of JAK/STAT pathway were first discovered in *Drosophila* (36). All three ligands, named unpaired (Upd), Upd2, and Upd3 bind the same receptor named Domelss (Dome) which shares sequence similarity with mammalian IL-6 receptor (37), but only Udp2 and Upd3 are induced by viral infection and provide protection from a viral infection (38). Notably, JAK/STAT pathway has been considered to be triggered in bystander cells rather than in infected cells, since *vir-1* was not induced in DCV-infected fat body and periovarian sheath, but was substantially induced in epithelial cells of the ventral epidermis or in the oviduct, in which no viral load was detected, suggesting that *vir-1* was induced after a signal generated by the DCV-infected cells (32).

The signaling pathways responsible for induction of mammalian cytokines may also give some clues to whether there exists any “cytokine” that transmits antiviral signals between insect cells. Viral nucleic acids in mammalian cells are recognized by diverse cytosolic RNA or DNA sensors, including toll-like receptors (TLRs), retinoic acid-inducible gene I (RIG-I), absent in melanoma 2 (AIM2), DNA-dependent activator of IFN-regulatory factors (DAI) and cyclic GMP-AMP synthase (cGAS) (39). The signal is eventually relayed to transcriptional factors, including interferon regulatory factor 3 (IRF3) and NF-κB *via* signaling adaptors, such as antiviral-signaling protein (MAVS) and stimulator of interferon genes (STING) to activate interferon expression (40, 41). Despite the fact that far less nucleic acids sensors have been identified in insects, STING-mediated antiviral immunity has been discovered in *Drosophila* and *Bombyx mori* recently (23, 42, 43). Epistatic analysis showed that dSTING acts upstream of IKKβ and NF-κB transcriptional factor Relish to regulate the expression of a set of antiviral molecules, including a putative transmembrane protein named Nazo. Flies bearing dSTING or Relish mutant displayed higher susceptibility to infection of DCV, VSV or Cricket paralysis virus (CrPV). Activation of Relish by BmSTING was also detected in silkworm cell as evidenced by the cleavage of Relish carboxy-terminal Ankyrin repetitive sequence, which releases Relish from sequestration in cytoplasm, when BmSTING was over-expressed. The evolutionary conservation in STING- and NF-κB-dependent antiviral signaling pathway between insects and mammals suggests functional similarity in their downstream effectors. Indeed, a few years ago an antiviral factor Vago which bears no sequence homology to mammalian cytokines was first reported to be induced in the fat body of flies upon DCV infection, later its mosquito homologs that may act like interferon have been identified in *Culex*, *Aedes* and *Anopheles* (44, 45). CxVago was produced and secreted by West Nile virus (WNV)-infected cells. Incubating naïve cells with supernatant collected from Vago-expressing cells activated the JAK/STAT signaling pathway and induced the expression of *vir-1* in naïve cells independent of Dome. A NF-κB binding site was identified in CxVago promoter region afterwards, and *Culex* Rel2 which is a *Drosophila* Relish homolog has been demonstrated to be required for induction of CxVago subsequently. In addition, activation of Rel2 upon SINV infection was observed in mosquito cells (46). Intriguingly, after incubating with supernatants harvested from cells expressing Relish activated form, naïve silkworm cells displayed substantial resistance to BmNPV infection. Certain polypeptides purified from the supernatants of DNV-infected mosquito cells also acted like cytokines, conferring antiviral activity to naïve cells (47, 48).

Interestingly, in both flies and mosquito cells, induction of Vago has been characterized to be Dicer-2 dependent, since Dicer-2-mutant flies or Dicer-2-silenced mosquito cells had significantly lower levels of Vago induced by viral infection compared to Dicer-2-intact controls. But mutation of other RNAi key players, such as Ago-2 and R2D2 had no impact on Vago expression, indicating that induction of Vago is independent of RNAi pathway. Phylogenetic analysis revealed Dicer-2, which is a key player in RNAi, is closely related to mammalian RIG-I in terms of their DExD/H-box helicase domain (49, 50). Both of them belong to RIG-I-like receptor (RLR) family along with some other cytoplasmic RNA sensors, including MDA5 and Laboratory of Genetics and Physiology 2 (LGP2). More recently, Dicer-2 has been reported to modulate viral DNA production *via* acting as a pattern recognition receptor similar to RLR that senses defective viral genomes (DVGs) (51). The absence of RIG-I proteins in insects but presence of the activity of RNA sensing and induction of antiviral factors which is carried out in a Dicer-2 dependent manner suggests Dicer-2 may be the archetypal RLR that activates the antiviral signaling pathway in insects. It is worth exploring whether Dicer-2, STING and Relish constitute a signaling axis that leads to the production of antiviral effectors and contributes to cell-cell communication.

Apparently, not all viral-induced molecules potentiate antiviral immunity, some may promote host survival by preventing immune signaling from over-activation. Diedel has been characterized as an immunomodulatory cytokine in *Drosophila* that was strongly induced following infection with slowly replicating viruses, such as SINV and VSV (52). *diedel* mutant flies developed persistent inflammation as a few immune-related genes, most of which are considered to be controlled by the IMD pathway, were up-regulated in the absence of viral infection. They also showed reduced survival after immune challenges without an increase in viral load, suggesting the IMD pathway which may contribute to viral-induced pathogenesis is required to be down-regulated. Interestingly, Diedel homologs have also been identified in the genome of three different and unrelated families of DNA viruses that infect Lepidoptera, including Entomopoxvirinae, Baculoviridae, and Ascoviridae (53). Transcriptome analysis found elevated expression of ascovirus *diedel* in infected *Spodoptera frugiperda* larvae (54), and expression of the ascovirus *diedel* partially rescued the reduced viability of *diedel* mutant flies (52). The possible horizontal transfer of immunomodulatory genes from host to virus represents a strategy that virus exploits to manipulate host immune response in favor of its own replication and dissemination.

### RNA Based Intercellular Communication: Transferring of Virus-Derived RNA Between Cells

Intercellular transferring of virus- or host-derived RNA, DNA and proteins from infected cells to neighboring cells are increasingly recognized as an important mean to mount a self-sustaining and even amplified innate immune response. Gap junctions, exosomes, microvesicles and plant plasmodesmata have been reported to deliver the substances originated from viral infected cells to immunize the other cells before arrival of the virus (55–58). Although the open circulatory system in insects is always believed to allow fast spread of virus in the hemolymph and migration beyond the primary site of replication, the possible cell-cell communication is supported by evidence of intercellular transferring of virus-derived RNA. Flies defective in dsRNA endocytosis or intracellular transport were hypersensitive to viral infection, and the high mortality was accompanied by hundredfold increase in viral titer, suggesting a systemic spread of dsRNA is required for antiviral immunity (59). Nanotube-like structures made of actin and tubulin were first reported in a study of the intercellular communication between *Drosophila* cells (60). Those membrane projections generated by viral-infected cells bridge neighboring cells for transferring of components of RNAi machinery, including Ago-2 and dsRNA between cells. A more recent study discovered that haemocytes acquire virus-derived dsRNA (vsRNA) by phagocytosing virus-infected cells and reverse-transcribe the viral RNA through endogenous transposon reverse transcriptases into DNA which serves as a template for transcription of secondary vsRNA in an Ago-2 dependent manner (61). The secondary vsRNA is secreted by haemocytes in exosome-like vesicles (ELVs) and spreads through the haemolymph. It is then processed into siRNA by cells taking up these ELVs and confers virus-specific immunity. Of note, this systemic antiviral potential of haemocyte-derived ELVs persists weeks after the onset of viral infection, thus it was proposed as an RNAi-based “adaptive immunity” in *Drosophila*.

## Discussion

Extracellular signaling network coordinates the systemic immune response through alarming or even arming the non-infected cells with messages from viral infected cells. Although it is one of the most important parts of immune response, much less have we learnt about the molecules or vesicles secreted by viral infected cells, ways to deliver them or the pathways they influence. Integrated omics approaches might be required to characterize the soluble substances in the fractionated extracellular fluid of viral infected cells in the future research, screening of target genes under regulation of signaling pathways that are activated by viral sensors would also help to narrow down the candidates. Furthermore, the absence of viral loads in tissues expressing antiviral marker genes (32) or passive protection of naïve flies against viral challenges conferred by injection of purified ELVs from viral infected flies (61) suggests a tissue-targeted delivery or diffusion throughout the entire body, therefore identification of molecules that act as receptors or carriers of those extracellular substances will decipher how the antiviral signal is transmitted between cells, which tissues or organs are targeted and which intracellular pathways are activated.

Although the lack of sequence similarity between insect and vertebrate cytokines impedes a sequence-function relationship analysis, the structural features they share suggest they are functionally related. For example, one subdomain of Diedel, consisting of an antiparallel β-sheet covered by an α-helix, resembles certain CC or CXC chemokine family members (62), which modulate immune response by maintaining proliferative homeostasis and attenuating apoptosis. Interestingly, recombinant human IL-8 was reported to promote the phagocytic activity of *Drosophila* S2 cells and enhance the expression of Upd-3 as well as some AMP genes, including *defensin*, *cecropin A1*, and *diptericin* (63), implying that certain membrane bound molecule may function as receptor to ligand that resembles the structure of IL-8.

Some danger signals, such as metabolites produced by viral-infected cells or damage-associated molecular pattern (DAMP) released by dead or damaged cells, may also serve as mediator for systemic inflammatory response. For instance, in mammalian models nitric oxide (NO) generated through NO synthase (NOS) which is upregulated upon viral infection can diffuse freely across cell membranes and activate antiviral mechanisms in various ways, including direct and indirect damage to viral genomes (64, 65). In insects, it is well documented that NO regulates immune response to bacteria, nematode and parasites characterized by AMP expression and melanin production (66–68), and a cell-based assay showed that NO inhibits DENV replication partly through suppressing RNA-dependent RNA polymerase (69), although its role in insect antiviral immunity has not been characterized. Actin, an evolutionarily-conserved DAMP was reported to selectivity induce JAK/STAT target genes through cytokine Upd3 in *Drosophila*, whether it confers antiviral activity needs further investigation (70).

Antiviral immune response induced by different viruses varies, which might be another factor that complicates the understanding of insect antiviral immunity. For instance, Vago/Vago-like expression was down-regulated upon the infection of virulent virus but not with avirulent virus in bumblebee (71). Fast replicating viruses, such as DCV, CrPV and Flock House virus (FHV), unlike slowly replicating viruses, did not induce Diedel expression (52). In the mosquito midgut, transcriptional level of Rel2 and its canonical target genes, such as *diptericin* and *attancin*, was not induced by DENV (72), but the activation of Rel2 was detected on protein level and knockdown of Rel2 significantly increased WNV viral load (44). The seemingly disagreement on the involvement of certain molecules in antiviral immunity suggests a careful assessment of their general or specific functions is required.

In addition to their potential roles in the antiviral immune response, some molecules also participate in the defense against other microbial challenges. For example, knock-down of dSTING resulted in more susceptibility to *Listeria* infection (73) and mutation of BmSTING led to defective autophagy of microsporidia in silkworm larvae (74), suggesting insect STING mediates immune signaling pathways in response to various pathogens. However, some cytokines that have been identified in insect immune defenses against bacteria or parasites, such as growth blocking peptide (GBP) which has been characterized as a cytokine switching humoral and cellular immune response (75, 76), and TNF ortholog Eiger which promotes apoptotic cell death *via* JNK pathway and aids clearance of extracellular pathogens (77, 78), are not reported in the antiviral response. Therefore, it will be interesting to investigate whether there exist multifaceted mediators in insect innate immunity.

While studies on the viral-induced intercellular communication are still preliminary in insects, they provide valuable insights into artificial manipulation of host immune response. Insulin/insulin-like peptide has been recently reported to potentiate JAK/STAT pathway *via* ERK to broadly inhibit flavivirus replication in fly and mosquito cells, and insulin-supplemented meal effectively reduced WNV titers in infected *Culex* mosquitos (79, 80). Although the change in insulin level induced by viral infection was not yet reported in insects or even linked to antiviral immunity prior to this report, the decrease in insulin secretion was found to be common in mammals after viral infection. Research efforts aimed at characterizing the intercellular communication will not only provide a greater depth of knowledge regarding extracellular signaling networks, but also potential targets for pest or disease control based on interfering intercellular communication or priming insects with molecules transmitting antiviral messengers.

## Author Contributions

The author confirms being the sole contributor of this work and has approved it for publication.

## Funding

This work is supported by grant of “National Natural Science Foundation of China” (No. 31672495) and Natural Science Foundation of Chongqing, China (cstc2020jcyj-msxmX0193).

## Conflict of Interest

The author declares that the research was conducted in the absence of any commercial or financial relationships that could be construed as a potential conflict of interest.

## References

[1] Yan N Chen ZJ. Intrinsic antiviral immunity. Nat Immunol (2012) 13:214–22. 10.1038/ni.2229 PMC3549670 22344284

[2] Schneider WM Chevillotte MD Rice CM. Interferon-stimulated genes: A complex web of host defenses. Annu Rev Immunol (2014) 32:513–45. 10.1146/annurev-immunol-032713-120231 PMC4313732 24555472

[3] Mussabekova A Daeffler L Imler J-L. Innate and intrinsic antiviral immunity in *Drosophila* . Cell Mol Life Sci (2017) 74:2039–54. 10.1007/s00018-017-2453-9 PMC5419870 28102430

[4] Lamiable O Imler J-L. Induced antiviral innate immunity in *Drosophila* . Curr Opin Microbiol (2014) 20:62–8. 10.1016/j.mib.2014.05.006 PMC4133299 24907422

[5] Kemp C Imler J-L. Antiviral immunity in *Drosophila* . Curr Opin Immunol (2009) 21:3–9. 10.1016/j.coi.2009.01.007 19223163 PMC2709802

[6] Hussain M Asgari S. MicroRNAs as mediators of insect host-pathogen interactions and immunity. J Insect Physiol (2014) 70:151–8. 10.1016/j.jinsphys.2014.08.003 25152509

[7] Asgari S. Role of microRNAs in insect host-microorganism interactions. Front Physiol (2011) 2:48:48. 10.3389/fphys.2011.00048 21886625 PMC3155871

[8] Wu Q Luo Y Lu R Lau N Lai EC Li W-X. Virus discovery by deep sequencing and assembly of virus-derived small silencing RNAs. Proc Natl Acad Sci USA (2010) 107:1606–11. 10.1073/pnas.0911353107 PMC2824396 20080648

[9] Petit M Mongelli V Frangeul L Blanc H Jiggins F Saleh MC. piRNA pathway is not required for antiviral defense in *Drosophila melanogaster* . Proc Natl Acad Sci USA (2016) 113:E4218–E27. 10.1073/pnas.1607952113 PMC4961201 27357659

[10] Tassetto M Kunitomi M Whitfield ZJ Dolan PT Sanchez-Vargas I Garcia-Knight M. Control of RNA viruses in mosquito cells through the acquisition of vDNA and endogenous viral elements. Elife (2019) 8:e41244. 10.7554/eLife.41244 31621580 PMC6797480

[11] Varjak M Leggewie M Schnettler E. The antiviral piRNA response in mosquitoes? J Gen Virol (2018) 99:1551–62. 10.1099/jgv.0.001157 30372405

[12] Lamiable O Arnold J de Faria I Olmo RP Bergami F Meignin C. Analysis of the contribution of hemocytes and autophagy to *Drosophila* antiviral immunity. J Virol (2016) 90:5415–26. 10.1128/jvi.00238-16 PMC4934735 27009948

[13] Nainu F Shiratsuchi A Nakanishi Y. Induction of apoptosis and subsequent phagocytosis of virus-infected cells as an antiviral mechanism. Front Immunol (2017) 8:1220. 10.3389/fimmu.2017.01220 29033939 PMC5624992

[14] Makino S Hamajima R Saito A Tomizaki M Iwamoto A Kobayashi M. *Bombyx mori* homolog of tumor suppressor p53 is involved in apoptosis-mediated antiviral immunity of *B. mori* cells infected with nucleopolyhedrovirus. Dev Comp Immunol (2018) 84:133–41. 10.1016/j.dci.2018.02.009 29448034

[15] Mocarski E Liu B Behura SK Clem RJ Schneemann A Becnel J. P53-mediated rapid induction of apoptosis conveys resistance to viral infection in *Drosophila melanogaster* . PloS Pathog (2013) 9:e1003137. 10.1371/journal.ppat.1003137 23408884 PMC3567152

[16] Settles EW Friesen PD. Flock house virus induces apoptosis by depletion of *Drosophila* inhibitor-of-apoptosis protein DIAP1. J Virol (2008) 82:1378–88. 10.1128/jvi.01941-07 PMC2224432 17989181

[17] Nainu F Tanaka Y Shiratsuchi A Nakanishi Y. Protection of insects against viral infection by apoptosis-dependent phagocytosis. J Immunol (2015) 195:5696–706. 10.4049/jimmunol.1500613 26546607

[18] Jiang L Liu W Guo H Dang Y Cheng T Yang W. Distinct functions of *Bombyx mori* peptidoglycan recognition protein 2 in immune responses to bacteria and viruses. Front Immunol (2019) 10:776. 10.3389/fimmu.2019.00776 31031766 PMC6473039

[19] Jiang L Goldsmith MR Xia Q. Advances in the arms race between silkworm and baculovirus. Front Immunol (2021). 10.3389/fimmu.2021.628151 PMC7900435 33633750

[20] Delorme-Axford E Klionsky DJ. Inflammatory-dependent sting activation induces antiviral autophagy to limit Zika virus in the *Drosophila* brain. Autophagy (2018) 15:1–3. 10.1080/15548627.2018.1539585 30354937 PMC6287683

[21] Liu Y Cherry S. Zika virus infection activates STING-dependent antiviral autophagy in the *Drosophila* brain. Autophagy (2018) 15:174–5. 10.1080/15548627.2018.1528813 PMC6287696 30260713

[22] Shelly S Lukinova N Bambina S Berman A Cherry S. Autophagy is an essential component of *Drosophila* immunity against vesicular stomatitis virus. Immunity (2009) 30:588–98. 10.1016/j.immuni.2009.02.009 PMC2754303 19362021

[23] Liu Y Gordesky-Gold B Leney-Greene M Weinbren NL Tudor M Cherry S. Inflammation-induced, STING-dependent autophagy restricts Zika virus infection in the *Drosophila* brain. Cell Host Microbe (2018) 24:57–68. 10.1016/j.chom.2018.05.022 29934091 PMC6173519

[24] Moy Ryan H Gold B Molleston Jerome M Schad V Yanger K Salzano M-V. Antiviral autophagy restricts Rift Valley fever virus infection and is conserved from flies to mammals. Immunity (2014) 40:51–65. 10.1016/j.immuni.2013.10.020 24374193 PMC3951734

[25] Nakamoto M Moy Ryan H Xu J Bambina S Yasunaga A Shelly Spencer S. Virus recognition by Toll-7 activates antiviral autophagy in *Drosophila* . Immunity (2012) 36:658–67. 10.1016/j.immuni.2012.03.003 PMC3334418 22464169

[26] Xu J Grant G Sabin Leah R Gordesky-Gold B Yasunaga A Tudor M. Transcriptional pausing controls a rapid antiviral innate immune response in *Drosophila* . Cell Host Microbe (2012) 12:531–43. 10.1016/j.chom.2012.08.011 PMC3479682 23084920

[27] Huang Z Kingsolver MB Avadhanula V Hardy RW. An antiviral role for antimicrobial peptides during the arthropod response to alphavirus replication. J Virol (2013) 87:4272–80. 10.1128/JVI.03360-12 PMC3624382 23365449

[28] Diamond MS Luplertlop N Surasombatpattana P Patramool S Dumas E Wasinpiyamongkol L. Induction of a peptide with activity against a broad spectrum of pathogens in the *Aedes aegypti* salivary gland, following infection with Dengue virus. PloS Pathog (2011) 7:e1001252. 10.1371/journal.ppat.1001252 21249175 PMC3020927

[29] Bao Y-Y Tang X-D Lv Z-Y Wang X-Y Tian C-H Xu YP. Gene expression profiling of resistant and susceptible *Bombyx mori* strains reveals nucleopolyhedrovirus-associated variations in host gene transcript levels. Genomics (2009) 94:138–45. 10.1016/j.ygeno.2009.04.003 19389468

[30] Merkling SH van Rij RP. Beyond RNAi: Antiviral defense strategies in *Drosophila* and mosquito. J Insect Physiol (2013) 59:159–70. 10.1016/j.jinsphys.2012.07.004 22824741

[31] Xu J Cherry S. Viruses and antiviral immunity in *Drosophila* . Dev Comp Immunol (2014) 42:67–84. 10.1016/j.dci.2013.05.002 23680639 PMC3826445

[32] Dostert C Jouanguy E Irving P Troxler L Galiana-Arnoux D Hetru C. The Jak-STAT signaling pathway is required but not sufficient for the antiviral response of drosophila. Nat Immunol (2005) 6:946–53. 10.1038/ni1237 16086017

[33] Majoros A Platanitis E Kernbauer-Hölzl E Rosebrock F Müller M Decker T. Canonical and non-canonical aspects of JAK–STAT signaling: lessons from interferons for cytokine responses. Front Immunol (2017) 8:29:29. 10.3389/fimmu.2017.00029 28184222 PMC5266721

[34] Morris R Kershaw NJ Babon JJ. The molecular details of cytokine signaling via the JAK/STAT pathway. Protein Sci (2018) 27:1984–2009. 10.1002/pro.3519 30267440 PMC6237706

[35] Schoggins JW Rice CM. Interferon-stimulated genes and their antiviral effector functions. Curr Opin Virol (2011) 1:519–25. 10.1016/j.coviro.2011.10.008 PMC3274382 22328912

[36] Zeidler MP Bausek N. The *Drosophila* JAK-STAT pathway. JAKSTAT (2014) 2:e25353. 10.4161/jkst.25353 PMC3772116 24069564

[37] Chen HW Chen X Oh SW Marinissen MJ Gutkins JS Hou SX. Mom identifies a receptor for the *Drosophila* JAK/STAT signal transduction pathway and encodes a protein distantly related to the mammalian cytokine receptor family. Gene Dev (2002) 16:388–98. 10.1101/gad.955202 PMC155335 11825879

[38] Kemp C Mueller S Goto A Barbier V Paro S Bonnay F. Broad RNA interference–mediated antiviral immunity and virus-specific inducible responses in *Drosophila* . J Immunol (2013) 190:650–8. 10.4049/jimmunol.1102486 PMC3538939 23255357

[39] Wu J Chen ZJ. Innate immune sensing and signaling of cytosolic nucleic acids. Annu Rev Immunol (2014) 32:461–88. 10.1146/annurev-immunol-032713-120156 24655297

[40] Liu S Cai X Wu J Cong Q Chen X Li T. Phosphorylation of innate immune adaptor proteins MAVS, STING, and TRIF induces IRF3 activation. Science (2015) 347:aaa2630. 10.1126/science.aaa2630 25636800

[41] Ran Y Shu H-B Wang Y-Y. MITA/STING: a central and multifaceted mediator in innate immune response. Cytokine Growth Factor Rev (2014) 25:631–9. 10.1016/j.cytogfr.2014.05.003 PMC7108248 24929887

[42] Hua X Li B Song L Hu C Li X Wang D. Stimulator of interferon genes (STING) provides insect antiviral immunity by promoting Dredd caspase–mediated NF-κB activation. J Biol Chem (2018) 293:11878–90. 10.1074/jbc.RA117.000194 PMC6066306 29875158

[43] Goto A Okado K Martins N Cai H Barbier V Lamiable O. The kinase IKKβ regulates a STING- and NF-κB-dependent antiviral response pathway in *Drosophila* . Immunity (2018) 49:225–34.e4. 10.1016/j.immuni.2018.07.013 30119996 PMC6267954

[44] Olson KE Paradkar PN Duchemin J-B Voysey R Walker PJ. Dicer-2-dependent activation of *Culex* Vago occurs via the TRAF-Rel2 signaling pathway. PloS Negl Trop Dis (2014) 8:e2823. 10.1371/journal.pntd.0002823 24762775 PMC3998923

[45] Paradkar PN Trinidad L Voysey R Duchemin JB Walker PJ. Secreted Vago restricts West Nile virus infection in *Culex* mosquito cells by activating the Jak-STAT pathway. Proc Natl Acad Sci USA (2012) 109:18915–20. 10.1073/pnas.1205231109 PMC3503207 23027947

[46] Avadhanula V Weasner BP Hardy GG Kumar JP Hardy RW. A novel system for the launch of alphavirus RNA synthesis reveals a role for the IMD pathway in arthropod antiviral response. PloS Pathog (2009) 5:e1000582. 10.1371/journal.ppat.1000582 19763182 PMC2738967

[47] Wang F Li X Hua X Xia Q. Screening and analysis of anti-BmNPV cytokines in silkworm (*Bombyx mori*). Sci Agricult Sinica (2018) 51:789–99. 10.3864/j.issn.0578-1752.2018.04.018

[48] Kanthong N Laosutthipong C Flegel TW. Response to Dengue virus infections altered by cytokine-like substances from mosquito cell cultures. BMC Microbiol (2010) 10:290. 10.1186/1471-2180-10-290 21078201 PMC2995469

[49] Deddouche S Matt N Budd A Mueller S Kemp C Galiana-Arnoux D. The DExD/H-box helicase Dicer-2 mediates the induction of antiviral activity in drosophila. Nat Immunol (2008) 9:1425–32. 10.1038/ni.1664 18953338

[50] Paro S Imler J-L Meignin C. Sensing viral RNAs by Dicer/RIG-I like ATPases across species. Curr Opin Immunol (2015) 32:106–13. 10.1016/j.coi.2015.01.009 PMC4336672 25658360

[51] Poirier EZ Goic B Tomé-Poderti L Frangeul L Boussier J Gausson V. Dicer-2-dependent generation of viral DNA from defective genomes of RNA viruses modulates antiviral immunity in insects. Cell Host Microbe (2018) 23:353–65.e8. 10.1016/j.chom.2018.02.001 29503180 PMC5857290

[52] Lamiable O Kellenberger C Kemp C Troxler L Pelte N Boutros M. Cytokine Diedel and a viral homologue suppress the IMD pathway in *Drosophila* . Proc Natl Acad Sci USA (2016) 113:698–703. 10.1073/pnas.1516122113 26739560 PMC4725508

[53] Gould A Mlih M Khericha M Birdwell C West AP. Karpac J. A virus-acquired host cytokine controls systemic aging by antagonizing apoptosis. PloS Biol (2018) 16:e2005796. 10.1371/journal.pbio.2005796 30036358 PMC6072105

[54] Zaghloul HAH Hice R Arensburger P Federici BA. Transcriptome analysis of the *Spodoptera frugiperda* ascovirus *in vivo* provides insights into how its apoptosis inhibitors and caspase promote increased synthesis of viral vesicles and virion progeny. J Virol (2017) 91:e00874–17. 10.1128/JVI.00874-17 PMC5686725 28956762

[55] Rosas-Diaz T Zhang D Fan PF Wang LP Ding X Jiang YL. A virus-targeted plant receptor-like kinase promotes cell-to-cell spread of RNAi. Proc Natl Acad Sci USA (2018) 115:1388–93. 10.1073/pnas.1715556115 PMC5819414 29363594

[56] Kalamvoki M Du T Roizman B. Cells infected with herpes simplex virus 1 export to uninfected cells exosomes containing STING, viral mRNAs, and microRNAs. Proc Natl Acad Sci USA (2014) 111:E4991–6. 10.1073/pnas.1419338111 PMC4246290 25368198

[57] Baroja-Mazo A Martin-Sanchez F Gomez AI Martinez CM Amores-Iniesta J Compan V. The NLRP3 inflammasome is released as a particulate danger signal that amplifies the inflammatory response. Nat Immunol (2014) 15:738–48. 10.1038/ni.2919 24952504

[58] Patel SJ King KR Casali M Yarmush ML. DNA-triggered innate immune responses are propagated by gap junction communication. Proc Natl Acad Sci USA (2009) 106:12867–72. 10.1073/pnas.0809292106 PMC2722330 19617563

[59] Saleh M-C Tassetto M van Rij RP Goic B Gausson V Berry B. Antiviral immunity in *Drosophila* requires systemic RNA interference spread. Nature (2009) 458:346–50. 10.1038/nature07712 PMC3978076 19204732

[60] Karlikow M Goic B Mongelli V Salles A Schmitt C Bonne I. *Drosophila* cells use nanotube-like structures to transfer dsRNA and RNAi machinery between cells. Sci Rep (2016) 6:27085. 10.1038/srep27085 27255932 PMC4891776

[61] Tassetto M Kunitomi M Andino R. Circulating immune cells mediate a systemic RNAi-based adaptive antiviral response in *Drosophila* . Cell (2017) 169:314–25.e13. 10.1016/j.cell.2017.03.033 28388413 PMC5730277

[62] Doucet D Coste F Kemp C Bobezeau V Hetru C Kellenberger C. Crystal structure of Diedel, a marker of the immune response of *Drosophila melanogaster* . PloS One (2012) 7:e33416. 10.1371/journal.pone.0033416 22442689 PMC3307722

[63] Malagoli D Sacchi S Ottaviani E. Unpaired (upd)-3 expression and other immune-related functions are stimulated by interleukin-8 in *Drosophila melanogaster* SL2 cell line. Cytokine (2008) 44:269–74. 10.1016/j.cyto.2008.08.011 18926718

[64] Abdul-Cader MS Amarasinghe A Abdul-Careem MF. Activation of toll-like receptor signaling pathways leading to nitric oxide-mediated antiviral responses. Arch Virol (2016) 161:2075–86. 10.1007/s00705-016-2904-x PMC7087267 27233799

[65] Nappi AJ Vass E Frey F Carton Y. Nitric oxide involvement in *Drosophila* immunity. Nitric Oxide (2000) 4:423–30. 10.1006/niox.2000.0294 10944427

[66] Eleftherianos I More K Spivack S Paulin E Khojandi A Shukla S. Nitric oxide levels regulate the immune response of *Drosophila melanogaster* reference laboratory strains to bacterial infections. Infect Immun (2014) 82:4169–81. 10.1128/Iai.02318-14 PMC4187883 25047850

[67] Foley E O’Farrell PH. Nitric oxide contributes to induction of innate immune responses to gram-negative bacteria in *Drosophila* . Gene Dev (2003) 17:115–25. 10.1101/gad.1018503 PMC195964 12514104

[68] Herrera-Ortiz A Martinez-Barnetche J Smit N Rodriguez MH Lanz-Mendoza H. The effect of nitric oxide and hydrogen peroxide in the activation of the systemic immune response of *Anopheles albimanus* infected with *Plasmodium berghei* . Dev Comp Immunol (2011) 35:44–50. 10.1016/j.dci.2010.08.004 20708028

[69] Takhampunya R Padmanabhan R Ubol S. Antiviral action of nitric oxide on dengue virus type 2 replication. J Gen Virol (2006) 87:3003–11. 10.1099/vir.0.81880-0 16963759

[70] Srinivasan N Gordon O Ahrens S Franz A Deddouche S Chakravarty P. Actin is an evolutionarily-conserved damage-associated molecular pattern that signals tissue injury in *Drosophila melanogaster* . Elife (2016) 5:e19662. 10.7554/eLife.19662 27871362 PMC5138034

[71] Niu J Meeus I Smagghe G. Differential expression pattern of Vago in bumblebee (*Bombus terrestris*), induced by virulent and avirulent virus infections. Sci Rep (2016) 6:34200. 10.1038/srep34200 27680717 PMC5040954

[72] Xi Z Ramirez JI Dimopoulos G. The *Aedes aegypti* Toll pathway controls dengue virus infection. PloS Pathog (2008) 4:e1000098. 10.1371/journal.ppat.1000098 18604274 PMC2435278

[73] Martin M Hiroyasu A Guzman RM Roberts SA Goodman AG. Analysis of *Drosophila* STING reveals an evolutionarily conserved antimicrobial function. Cell Rep (2018) 23:3537–50.e6. 10.1016/j.celrep.2018.05.029 29924997 PMC6114933

[74] Hua X Xu W Ma S Xia Q. STING-dependent autophagy suppresses *Nosema bombycis* infection in silkworms, *Bombyx mori* . Dev Comp Immunol (2020) 115:103862. 10.1016/j.dci.2020.103862 32916206

[75] Ishii K Hamamoto H Kamimura M Nakamura Y Noda H Imamura K. Insect cytokine paralytic peptide (PP) induces cellular and humoral immune responses in the silkworm *Bombyx mori* . J Biol Chem (2010) 285:28635–42. 10.1074/jbc.M110.138446 PMC2937889 20622022

[76] Tsuzuki S Matsumoto H Furihata S Ryuda M Tanaka H Jae Sung E. Switching between humoral and cellular immune responses in *Drosophila* is guided by the cytokine GBP. Nat Commun (2014) 5:4628. 10.1038/ncomms5628 25130174 PMC5755975

[77] Schneider DS Ayres JS Brandt SM Costa A Dionne MS Gordon MD. *Drosophila* eiger mutants are sensitve to extracellular pathogens. PloS Pathog (2007) 3:e24. 10.1371/journal.ppat.0030041 17381241 PMC1829408

[78] Igaki T Kanda H Yamamoto-Goto Y Kanuka H Kuranaga E Aigaki T. Eiger, a TNF superfamily ligand that triggers the *Drosophila* JNK pathway. EMBO J (2002) 21:3009–18. 10.1093/emboj/cdf306 PMC126061 12065414

[79] Trammell CE Goodman AG. Emerging mechanisms of insulin-mediated antiviral immunity in *Drosophila melanogaster* . Front Immunol (2019) 10:2973:2973. 10.3389/fimmu.2019.02973 31921210 PMC6934001

[80] Ahlers LRH Trammell CE Carrell GF Mackinnon S Torrevillas BK Chow CY. Insulin potentiates JAK/STAT signaling to broadly inhibit flavivirus replication in insect vectors. Cell Rep (2019) 29:1946–60.e5. 10.1016/j.celrep.2019.10.029 31722209 PMC6871768

